# Effect of eliminating CD4-count thresholds on HIV treatment initiation in South Africa: An empirical modeling study

**DOI:** 10.1371/journal.pone.0178249

**Published:** 2017-06-15

**Authors:** Jacob Bor, Shahira Ahmed, Matthew P. Fox, Sydney Rosen, Gesine Meyer-Rath, Ingrid T. Katz, Frank Tanser, Deenan Pillay, Till Bärnighausen

**Affiliations:** 1 Department of Global Health, Boston University School of Public Health, Boston, MA, United States of America; 2 Africa Health Research Institute, Somkhele and Durban, South Africa; 3 Health Economics and Epidemiology Research Office, University of Witwatersrand, Johannesburg, South Africa; 4 Department of Epidemiology, Boston University School of Public Health, Boston, MA, United States of America; 5 Division of Women’s Health, Brigham and Women’s Hospital, Boston, MA, United States; 6 Center for Global Health, Massachusetts General Hospital, Harvard Medical School, Boston, MA, United States; 7 Harvard Medical School, Boston, MA, United States; 8 Department of Virology, University College London, London, United Kingdom; 9 Heidelberg Institute of Public Health, University of Heidelberg, Heidelberg, Germany; 10 Department of Global Health and Population, Harvard T.H. Chan School of Public Health, Boston, MA, United States of America; Ghent University, BELGIUM

## Abstract

**Background:**

The World Health Organization recommends initiating antiretroviral therapy (ART) regardless of CD4 count. We assessed the effect of ART eligibility on treatment uptake and simulated the impact of WHO’s recommendations in South Africa.

**Methods:**

We conducted an empirical analysis of cohort data using a regression discontinuity design, and then used this model for policy simulation. We enrolled all patients (n = 19,279) diagnosed with HIV between August 2011 and December 2013 in the Hlabisa HIV Treatment and Care Programme in rural South Africa. Patients were ART-eligible with CD4<350 cells/mm^3^ or Stage III/IV illness. We estimated: (1) distribution of first CD4 counts in 2013; (2) probability of initiating ART ≤6 months of HIV diagnosis under existing criteria at each CD4 count; (3) probability of initiating ART by CD4 count if thresholds were eliminated; and (4) number of expected new initiators if South Africa eliminates thresholds.

**Findings:**

In 2013, 38.9% of patients diagnosed had a CD4 count ≥500. 8.0% of these patients initiated even without eligible CD4 counts. If CD4 criteria were eliminated, we project that an additional 19.2% of patients with CD4 ≥500 would initiate ART; 72.8% would not initiate ART despite being eligible. Eliminating CD4 criteria would increase the number starting ART by 26.7%. If these numbers hold nationally, this would represent an additional 164,000 initiators per year, a 5.2% increase in patients receiving ART and 5.3% increase in programme costs.

**Conclusions:**

Removing CD4 criteria alone will modestly increase timely uptake of ART. However, our results suggest the majority of newly-eligible patients will not initiate. Improved testing, linkage, and initiation procedures are needed to achieve 90-90-90 targets.

## Background

In September 2015, the World Health Organization (WHO) recommended that all adults living with HIV initiate ART regardless of CD4 cell count or WHO clinical stage[1]. These guidelines were issued with the goal of expanding the numbers of HIV-infected persons on therapy, reducing onward transmission[2], and decreasing morbidity and mortality among infected people [3][4].

Despite the considerable enthusiasm for immediate initiation of ART, many questions remain regarding its implications in high-burden countries. Expanding ART eligibility will almost surely lead to an increase in ART uptake. Yet not all patients with ART-eligible CD4 counts go on to initiate therapy. About one third are lost to initiation[5–9], and loss may be even higher in asymptomatic patients presenting at high CD4 counts[10]. Still other patients initiated under the prior guidelines because of staging criteria (stage III/IV HIV illness) or pregnancy and would not have been impacted by elimination of CD4 thresholds. As countries implement the 2015 WHO guidelines[11], understanding their likely impact on ART uptake is critical for program design and budgetary projections. Understanding the impact of eliminating eligibility barriers will also inform the next wave of interventions to increase treatment coverage and to achieve global targets of 90% tested, 90% initiated, and 90% virally suppressed[1].

Existing evidence on ART uptake in patients eligible at lower CD4 counts may not provide reliable guidance on what to expect under universal eligibility. Patients presenting at lower CD4 counts may have greater need for ART and may be more likely to initiate. On the other hand, patients presenting at higher CD4 counts may face fewer obstacles to seeking care. Randomized trials such as START, TEMPRANO, and HPTN-052 systematically exclude patients with low motivation to start ART during screening procedures and actively track those who default from care and therefore do not provide evidence on the real world impact of immediate eligibility for ART. To date, little is known about how patients presenting for care at high CD4 counts will respond to being eligible for ART in sub-Saharan Africa. Although simulation studies have projected that test-and-treat could reduce HIV transmission and alter the course of the epidemic [12], these simulations are based on a wide range of assumptions about ART uptake under HIV test-and-treat and not on real world causal evidence. Our study aims to fill this gap, identifying the impact of universal ART eligibility on rates of ART initiation.

South Africa has the highest HIV burden worldwide, with an estimated 6.5 million adults infected with HIV and more than 3 million people on treatment[13]. National guidelines extended ART eligibility to all patients with CD4 counts < 350 cells/mm^3^ in August 2011 (from the previous 200-cell threshold) and to patients with CD4 < 500 in January 2015[14–16]. South Africa adopted WHO recommendations to eliminate CD4 criteria in September 2016[11].

In this paper, we use data from a large cohort of patients who sought HIV care in the public sector in KwaZulu-Natal Province, South Africa to estimate the impact of immediate eligibility for all HIV patients at diagnosis, regardless of CD4 count, on treatment uptake. We use a regression discontinuity design to compare rates of ART uptake in patients presenting with CD4 counts that were eligible vs. ineligible for ART based on a prior threshold. These estimates offer some guidance on the likely impact of eliminating CD4 count thresholds on numbers of new ART initiators if no other changes are made to HIV testing, linkage to care, or treatment initiation procedures.

## Methods

### Ethics

Ethical approvals for data collection were obtained by the Africa Health Research Institute from the Biomedical Research Ethics Committee of the University of KwaZulu-Natal, South Africa. This study consisted of secondary analysis of de-identified data and was determined to be “not human subjects research” by the Boston University Medical Campus IRB.

### Data and study population

We included all patients presenting for HIV care August 12, 2011 –December 31, 2013 in the Hlabisa HIV Treatment and Care Programme (Hlabisa Cohort)[17], the public-sector ART program serving Hlabisa sub-district, KwaZulu-Natal, South Africa. This program, extensively described[18–20], follows South African national guidelines for HIV care and is located in the province with the highest HIV prevalence nationally. Initiation dates were obtained from clinical charts, and dates and values of CD4 counts obtained from the country’s National Health Laboratory Service. CD4 blood samples are typically drawn the same day a patient tests positive for HIV and first test date was interpreted as date of HIV diagnosis. After the CD4 count was drawn, the specimen was sent to a centralized laboratory. Patients were expected to return for their results one week later and if ART-eligible, to complete three treatment literacy and readiness counseling sessions prior to initiation[17].

Patients were included in the analysis from the date of their first CD4 count and followed through December 31, 2013, regardless of whether they initiated ART. Patients were excluded if they never had a CD4 count or if their first CD4 count occurred after ART initiation. Patients were eligible for ART with CD4<350 or WHO Stage III/IV HIV illness. When assessing six months ART uptake, we excluded patients with a first CD4 count after June 30, 2013 to ensure adequate follow-up. We made no exclusions for pregnant women, TB patients, or individuals presenting with Stage III/IV illness, who would have been eligible for ART regardless of whether their CD4 count was below 350 cells. Thus, our results have an intention-to-treat interpretation, illuminating the aggregate effect of eliminating CD4 thresholds for all patients seeking care.

The primary outcome was whether the patient initiated ART within six months after the patient was diagnosed with HIV (first CD4 count). The presence of a CD4 count signifies the lab test was conducted and does not imply the patient received the result or was staged. Failure to initiate ART thus may reflect clinical attrition at any point in the lead up to ART initiation or the active decision of a patient or provider not to start ART[6,21]. The key exposures were the value of a patient’s CD4 count and the policy regime in effect at the time of presentation. CD4 counts were top-coded at 999 due to sparseness in the data beyond 1000 cells/mm^3^.

We considered ART uptake under three policy regimes. Under pre-2015 South African guidelines (*350-cell regime*), all patients presenting <350 were eligible to initiate ART and patients presenting ≥350 were eligible only if they had Stage III/IV HIV illness. Our data come from this period; however, they allow us to simulate ART uptake under later regimes. Under South Africa’s January 2015 guideline revision (*500-cell regime*), all patients with CD4 counts <500 were eligible; those over 500 were eligible only on the basis of a Stage III/IV condition. Under 2015 WHO guidelines (*no threshold regime*), which South Africa adopted in September 2016, all patients would be considered eligible regardless of CD4 count. At the time of writing, data were not available for patients presenting in Hlabisa after South Africa switched to a 500-cell threshold. In the future, data from these latter periods can be used to assess the accuracy of our projections.

### Study design and procedures

#### A potential outcomes model of ART uptake

The effect of raising the CD4 threshold from (a) 350 to 500 and (b) 500 to no threshold on the number of new initiators can be stated mathematically as:

$$\#NewInitiators=\int_{350}^{499}f_{CD4}*\{f_{ART(1)|CD4}-f_{ART(0)|CD4}\}dc$$ (a)

$$\#NewInitiators=\int_{500}^{\infty }f_{CD4}*\{f_{ART(1)|CD4}-f_{ART(0)|CD4}\}dc$$ (b)

These equations can be understood as follows: at each CD4 count 350–499 or over 500, the number of patients who will initiate because of the guideline change is equal to the number of patients presenting with a given CD4 count (*f*_*CD4*_), multiplied by the impact of eligibility on ART uptake at that CD4 count, (*f*_*ART(1)|CD4*_ *–f*_*ART(0)|CD4*_). This latter quantity is the difference–at each CD4 count–between the probability a patient initiates ART under a policy regime in which that CD4 count is ART-eligible (*f*_*ART(1)|CD4*_) and the probability that a patient initiates ART under a policy regime in which that CD4 count is not ART-eligible (*f*_*ART(0)|CD4*_). The aggregate impact of the policy is obtained by integrating across the range of CD4 counts affected, [350,499] and [500,∞).

#### Estimation of model parameters

**Fig 1:**
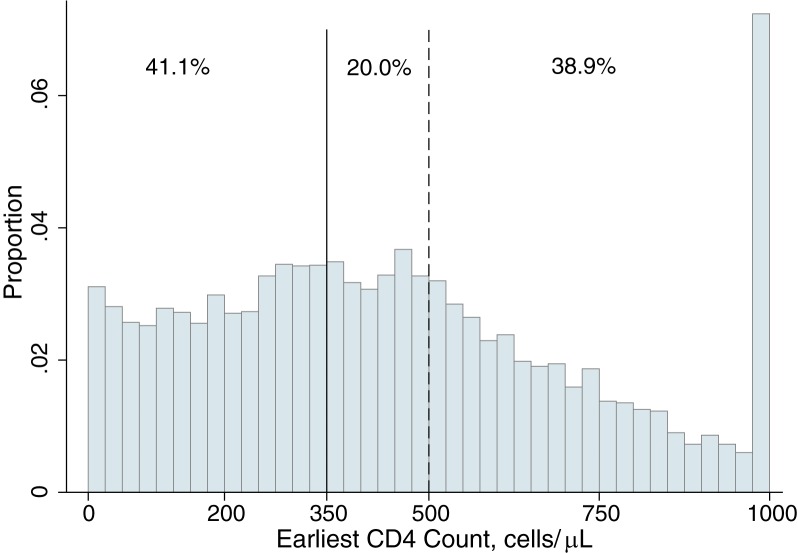
Distribution of earliest CD4 counts among patients seeking care for HIV in 2013. Data include all patients presenting to the Hlabisa HIV Treatment and Care Programme, the public sector ART program serving Hlabisa sub-district, rural KwaZulu-Natal. Data are shown for 2013, the last complete year for which data were available, and are top-coded at 999 cells. Under the assumption that the 2013 distribution of first CD4 counts reflects the future distribution among patients seeking care, the area under the curve between 350 and 500 shows the proportion of patients who would be expected to be newly eligible based on the January 2015 guideline revision. The proportion to the right of 500 shows the additional proportion of patients who would be expected to be eligible if South Africa adopts September 2015 WHO recommendations to initiate all patients regardless of CD4 count.

To estimate *f*_*CD4*_, we assessed the distribution of CD4 counts at time of clinical presentation in 2013, the most recent year for which data were available. We present the full distribution as Fig 1 and report the proportions of patients presenting with CD4 counts <350, 350–499, and ≥500.

**Fig 2:**
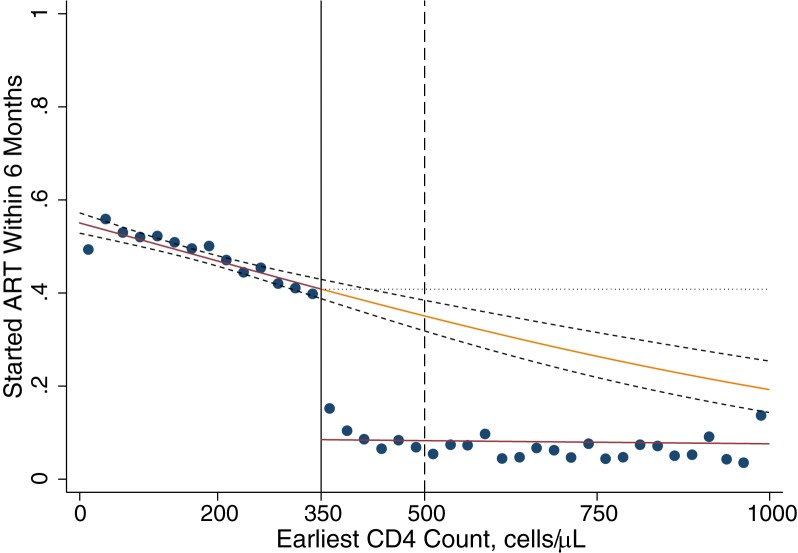
Proportion of patients starting ART within 6 months, by earliest CD4 count. Graph plots the probability of starting ART conditional on CD4 count, as observed in patients with a first CD4 count 12 August 2011–30 June 2012. Each dot is the binned average within a range of CD4 cells: 0–24, 25–49,…,975–999. Fitted regression lines are from a logistic regression model, allowing for separate slopes on either side of the threshold and an intercept shift at the threshold. The orange line displays the predicted (extrapolated) probabilities of initiation that would be expected if all patients were eligible for ART regardless of CD4 count. 95% confidence intervals are shown for the regression fit. The area under the red lines reflects the proportion of patients initiating under 2011–2014 guidelines. The area to the right of the 350 threshold, between the orange line and red line reflects the proportion of patients who would be expected to initiate under eligibility expansions to 500 and to all CD4 counts. The dotted horizontal line illustrates the upper bound used in sensitivity analysis, assuming that if eligible ART uptake above 350 was equal to ART uptake observed at 350.

The function *f*_*ART(0)|CD4*_ represents the probability of initiating ART if a patient’s CD4 count is not ART-eligible, i.e. the probability of initiating ART for other reasons. To estimate *f*_*ART(0)|CD4*_ for patients presenting with CD4 ≥350, we used our observed data collected during the 350-cell policy regime. While patients presenting ≥ 350 cells did not have eligible CD4 counts, some initiated anyway because of WHO staging criteria or because they were pregnant. We estimated the *f*_*ART(0)|CD4*_ among those with CD4 ≥350 using logistic regression (Fig 2).

The function *f*_*ART(1)|CD4*_ denotes the probability of initiating ART if a person’s CD4 count is eligible. The observed data provide insight on *f*_*ART(1)|CD4*_ for patients presenting with CD4 <350, who were ART-eligible under the extant policy regime, but *f*_*ART(1)|CD4*_ is not directly observed ≥350. To predict what would happen if eligibility were extended to patients ≥350, we used logistic regression to model *f*_*ART(1)|CD4*_ in patients with CD4 <350 and extrapolated our model predictions to patients with CD4 counts ≥350, as shown in Fig 2 and described below.

Our analytic approach is based on a regression discontinuity design, a quasi-experimental approach used to assess causal effects when a treatment is assigned based on a threshold rule. We fit a logistic regression model to the complete range of the data, allowing for an intercept shift at the eligibility threshold and for separate slopes on either side of the threshold, and estimated predicted probabilities at every integer CD4 count. Depending on the patient’s CD4 count, these predicted probabilities describe the likelihood of initiating ART under one of two counterfactual states of the world: *f*_*ART(1)|CD4*_ if eligible and *f*_*ART(0)|CD4*_ if not eligible. Visual inspection revealed good fit relative to binned averages. Extrapolating the “if eligible” regression line (*f*_*ART(1)|CD4*_), we forecast the probability that a patient with a first CD4 count above 350 would have initiated ART within six months had they, counter-to-fact, presented for care during the *500-cell* or *no threshold* regimes. 95% confidence intervals for the extrapolated line were estimated to account for uncertainty in the logistic regression parameters.

Our study differs from the typical “local” implementation of regression discontinuity, which emphasizes causal effects “at the threshold”[8,22–25]. In the local design, noise in measured CD4 counts ensures that patients are similar just above and just below the threshold, such that treatment is as good as randomly assigned in a small neighborhood around the threshold [8,26]. The local approach has strong internal validity but is not generalizable away from the threshold if the treatment effect varies with the assignment variable.

Because our goal was to simulate the impact of large changes in the eligibility threshold, we opted instead for a “global” regression discontinuity design, which enables inferences on *f*_*ART(1)|CD4*_ and *f*_*ART(0)|CD4*_ away from the threshold under additional assumptions. Inferences based on the extrapolation of *f*_*ART(1)|CD4*_ are valid under the assumption that the functional form of the predicted initiation probabilities in the unobserved ranges are correctly specified based on data in the observed range. This is a strong and untestable assumption, but may be satisfied if–as in this analysis–the functional form is simple, fits the data well in the observed range, and is justified for biological or behavioral reasons. It is unlikely, for example, that the probability of starting ART among eligible patients increases substantially at higher CD4 counts. Because this approach is sensitive to the accuracy of the extrapolation of *f*_*ART(1)|CD4*_, we also consider, as a plausible upper bound, a scenario in ART uptake was constant above 350 cells, *f*_*ART(1)|CD4>350*_ = *f*_*ART(1)|CD4 = 350*_.

The global regression discontinuity design was one of the original implementations of regression discontinuity, described by Rubin in 1977 [27], and represents one of several approaches to identification that has been used in regression discontinuity designs [26]. For completeness, we also implemented a “local” regression discontinuity design to estimate the causal effect of eligibility on ART uptake at the 350-cell threshold, using local linear regression models[28] with a data-driven optimal bandwidth[29]. We note that, though internally valid under weaker assumptions, this local approach estimates a parameter–the effect at the threshold, *f*_*ART(1)|CD4 = 350*_*—f*_*ART(0)|CD4 = 350*_ *–*which differs from the estimand of interest and may differ in practice due to effect heterogeneity. Additionally, in our example, the local effect may be attenuated by eligibility crossover among patients initially presenting just above the threshold and later reassessed with a CD4 count below 350.

#### Simulated effects of raising CD4 thresholds

**Fig 3:**
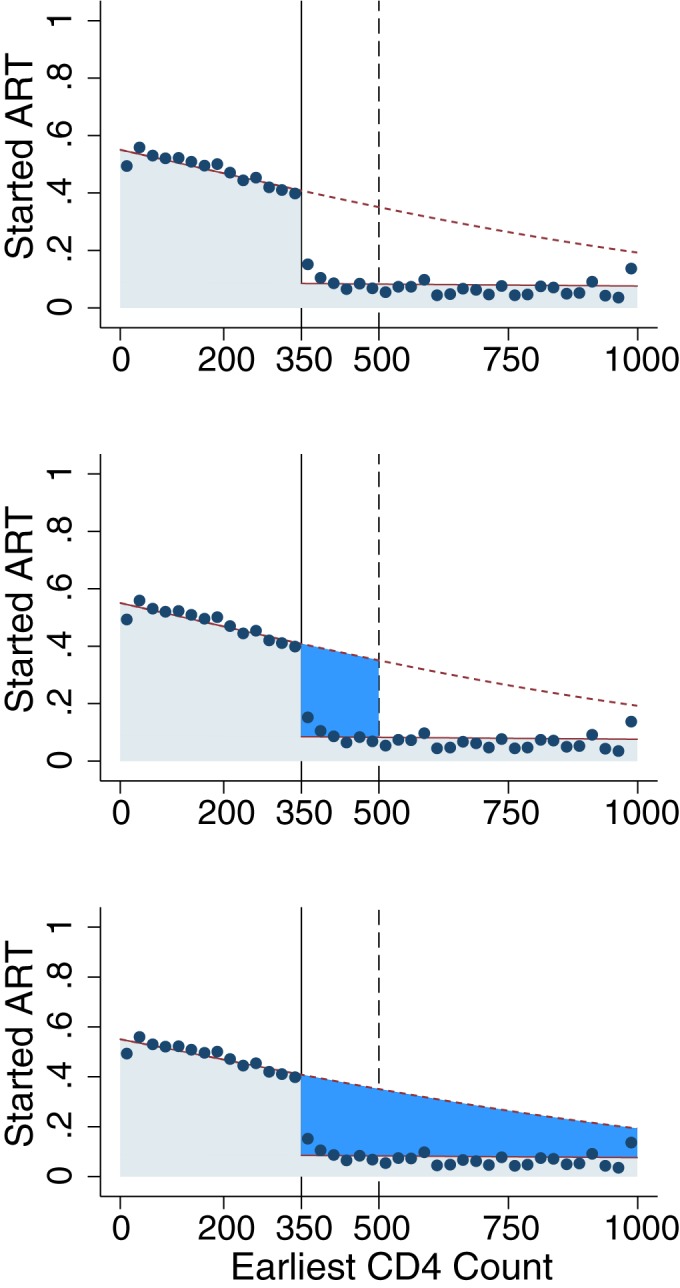
Projected impact of eligibility expansions on the proportion starting ART. Each dot is the binned average within a range of CD4 cells: 0–24, 25–49,…,975–999. Fitted regression lines are from a logistic regression model, allowing for separate slopes on either side of the threshold and an intercept shift at the threshold. The dotted line displays the predicted (extrapolated) probabilities of initiation that would be expected if all patients were eligible for ART regardless of CD4 count. The area under the solid lines reflects the proportion of patients initiating under 2011–2014 guidelines. The area to the right of the 350 threshold, between the dashed line and solid line reflects the proportion of patients who would be expected to initiate under eligibility expansions to 500 and to all CD4 counts.

We used our estimates of the functions *f*_*CD4*_, *f*_*ART(1)|CD4*_, *and f*_*ART(0)|CD4*_ to model the effect of (a) extending eligibility to patients presenting with CD4 350–500 and (b) extending eligibility to patients presenting with CD4 ≥500, as depicted in Fig 3. We simulated the following quantities (formulas for 350–500 not shown):

(1) Percent of patients with a first CD4 count in each range in 2013: i.e., $\int_{500}^{\infty }f_{CD4}dc$
(2) Percent of patients in each range who would have initiated ART even under the old regime, due to WHO stage or provider decision: i.e., $\int_{500}^{\infty }f_{CD4}*f_{ART(0)|CD4}dc$
(3) Percent of patients in each range expected to initiate ART under expanded CD4 criteria, i.e. $\int_{500}^{\infty }f_{CD4}*f_{ART(1)|CD4}dc$
(4) Percent of patients in each range expected to initiate ART as a result of expanded CD4 criteria, i.e. “new initiators”, (3)–(2)
(5) Percent of patients in each range expected not to initiate ART in spite of being eligible under expanded criteria, i.e. 100%–(3)
(6) Ratio of “new initiators” to the number of ART initiators observed in 2013, expressed as a percent increase in the number of ART initiators anticipated due to eligibility expansion

Generalizing from Hlabisa to the whole of South Africa requires strong assumptions. And yet, it is useful to put these results in perspective. Leveraging national data on the number of new ART initiators and the number remaining on ART and an existing model of the cost of the national ART programme, the National ART Cost Model (NACM)[30], we additionally projected:

(7) Number of additional ART initiators per year expected nationally due to the policy change, estimated by multiplying the ratio derived in (6) by the number of initiators in 2013 nationally, 614,000 (National Department of Health, District Health Information System).
(8) Percent increase (per year) in the total number of South Africans receiving ART, a critical parameter to model the added prevention benefit of early ART eligibility. We estimated this parameter as the number calculated in (7) divided by 3 million, the approximate number of people receiving ART in South Africa in 2015.
(9) Finally, we projected the incremental cost to the national ART programme as a result of this additional uptake over the next 5 financial years.

## Results

As shown in Table 1, 19,279 patients presented for HIV care and treatment services in the Hlabisa HIV Treatment and Care Programme and had a first CD4 count between August 12, 2011 and December 31, 2013. Of these, 15,038 had a first CD4 count between 12 August 2011 and 30 June 2013, and were included in our regression sample. Age and sex were similar in the full sample and regression sample, with 68.5% and 68.4% female and a median age of 30.2 and 29.9 respectively. Patients in the regression sample presented with a median CD4 count of 332 cells/mm^3^, compared to 354 in the full sample.

**Table 1:** Characteristics of the sample.

Parameter	Full sample^a^	Regression sample^b^
Patients presenting for care; n	19,279	15,038
Female; % (n)	68.5% (12,902)	68.4% (10 105)
Age in years; median (IQR)	30.2 (24.0, 38.7)	29.9 (23.8, 38.5)
First CD4 count; median (IQR)	354 (187, 555)	332 (174, 520)
Started ART within 6 months; % (n)	25.0% (4827)	29.2% (4393)
Started ART by Dec 2013; % (n)	27.6% (5330)	32.6% (4896)

^a^Full sample includes all patients presenting for HIV care and treatment services in the Hlabisa HIV Treatment and Care Programme from August 12 2011, when South Africa adopted the 350-cell initiation threshold, to December 31 2013, the last date when data on ART start dates were systematically entered into the database.

^b^The regression sample is limited to patients with a first CD4 count August 12 2011 through June 30 2013, to ensure a full six months of follow-up to assess ART uptake. Data on sex were missing for 445 patients and on age for 432 patients; summary statistics are among non-missing.

Fig 1 displays the distribution of first CD4 counts among patients clinically diagnosed with HIV in 2013. Of the 7973 patients diagnosed in 2013, 41.1% presented with CD4 counts below 350 cells/mm^3^, 20.0% presented between 350 and 500 cells/mm^3^, and 38.9% presented above 500 cells/mm^3^.

Fig 2 plots ART initiation probabilities against the value of a patient’s first CD4 count. The predictions from our logistic regression model (coefficients reported in S1 Table) closely track the raw data, plotted as binned averages. As would be expected, patients presenting for care with CD4 counts below the 350-cell threshold were more likely to start ART within six months than patients presenting above the threshold. For patients presenting with CD4 counts above the 350-cell threshold, the probability of starting ART was about 8% and varied little with CD4 count; these patients were presumably initiated on the basis of a Stage III/IV HIV illness. For patients presenting below the threshold, the overall probability of starting ART was 47.8%. Within this group, the value of the patient’s first CD4 count was strongly correlated with the probability of initiating ART, with healthier (higher CD4 count) patients substantially less likely to start than sicker patients. The effect of the policy change is modeled as the difference between the extrapolated predictions for ART initiation *if eligible* (orange line in Fig 2) and the predictions based on the observed data for patients above 350 who did not have ART-eligible CD4 counts (red line in Fig 2), weighted by the distribution seeking care shown in Fig 1.

Fig 3 shows how policies raising the threshold from 350 to 500 or eliminating the threshold would affect patients presenting at different CD4 counts. The blue shaded region illustrates the proportion of patients expected to start ART within six months under each scenario. The area above the blue region–between the regression line and 100%–shows the proportion of patients who do not start ART within six months.

Our quantitative estimates of the impact of eligibility expansion on the Hlabisa ART programme are shown in Table 2. Among patients presenting with CD4≥500, 27.2% are expected to initiate ART within six months with the elimination of CD4 criteria. We anticipate that 8.0% of patients with CD4≥500 would have initiated ART even under the old regime, e.g. due to staging, implying that 29% (8.0/27.2) of patients expected to initiate with CD4≥500 would have initiated regardless of eligibility expansion. The effect of eliminating CD4 thresholds on new initiators will be to increase the percent initiating ART from 8.0% to 27.2%, a difference of 19.2 percentage points (95% CI 13.6 to 21.1) among those patients presenting with CD4≥500. Even with CD4 criteria eliminated, 72.8% of patients with CD4≥500 are expected not to initiate ART despite being eligible, unless there are further changes to initiation protocols (Table 2, Fig 2, Fig 3). Qualitatively similar results are observed for an increase in the CD4 threshold from 350 to 500 cells (Table 2).

**Table 2:** Projected impact of eligibility expansions on numbers of new ART initiators.

Parameter	First CD4 count 350 to 500 cells	First CD4 count at least 500 cells	
*Distribution of first CD4 counts in Hlabisa Cohort*			
(1) Percent of all patients seeking care in 2013	20.0%	38.9%	
*Predicted ART uptake in patients presenting with CD4 350–500 and* ≥*500 cells in Hlabisa Cohort*			
(2) Percent expected to initiate ART even under the old regime, e.g. due to staging	8.4%		8.0%
(3) Percent expected to initiate ART under expanded CD4 criteria	37.9% (35.3 to 40.7)		27.2% (20.3 to 27.8)
(4) Percent expected to initiate ART due to expanded CD4 criteria, i.e. “new initiators”, (3)–(2)	29.5% (26.9 to 32.3)		19.2% (13.6 to 21.1)
(5) Percent expected not to initiate ART in spite of being eligible under expanded criteria, 100%–(4)	62.1% (60.3 to 64.7)		72.8% (72.2 to 79.7)
(6) Percent increase: ratio of “new initiators” to ART initiators in 2013, (4) * 7973/2233–100%	21.2% (19.3 to 23.2)		26.7% (18.9 to 29.3)
*National projections*			
(7) Number of additional ART initiators per year nationally expected from expanded CD4 criteria, (6) * 617,000	130,000 (119,000 to 142,000)		164,000 116,000 to 180,000)
(8) Percent increase in South Africans receiving ART due to expanded CD4 criteria, (7) / 3 million	4.3% (4.0 to 4.7)		5.2% (3.7 to 5.8)
(9) Incremental cost (per year) and percent increase in total cost of South African ART programme due to expanded criteria (averaged 2016/17-2020/21)			$42 million 5%

Table presents projected impacts of guideline changes extending eligibility for ART to all patients presenting with CD4 counts 350 to 500 cells/mm^3^ and over 500 cells/mm^3^. Procedures for calculations are described in text. There were 7973 patients who sought care and 2233 ART initiators in 2013 in the Hlabisa cohort. National projections based on 614,000 ART initiators in 2013 and 3 million people currently receiving ART; national cost calculations for the elimination of CD4 thresholds were based on the National ART Cost Model (NACM), averaged over five years– 2016/17 to 2020/21.

Raising the CD4 threshold to 500 and eliminating CD4 criteria entirely were projected to increase the annual number of initiators by 21.2% and 26.7%, respectively, in Hlabisa. If we scale the Hlabisa results by the number of ART initiators nationally in 2013 (n = 614,000), we project that these expansions would lead to increases of 130,000 and 164,000 patients, respectively. These numbers represent increases of 4.3% and 5.2% in the total population of patients receiving ART in South Africa (Table 2).

In sensitivity analysis, assuming constant uptake above 350 cells, we estimated that 40.8% of patients would initiate ART under revised guidelines (S2 Table, Fig 2). In patients presenting ≥500 cells/mm^3^, eliminating thresholds would lead an additional 32.8% of patients to initiate ART, representing a 45.7% increase over baseline initiation levels, higher than the 26.7% estimated in our primary specification. Nationally, this would translate into 280,000 initiators per year and a 9.3% annual increase in numbers receiving ART (S2 Table). We caution that these numbers should be treated as an upper bound as it is unlikely that the strong downward sloping trend in the likelihood of starting ART would simply flat-line above 350 cells. Still, even in this scenario, 59.2% of patients presenting for HIV care ≥500 cells would be expected not to initiate ART within six months despite being eligible (S2 Table).

The results of the local regression discontinuity approach were broadly consistent with our global estimates (S1 Fig, S1 Table). The local linear model better captured non-linearities just above the threshold (likely due to crossover into eligibility) and thus estimated a smaller gap at the threshold– 22.9 vs. 32.1 percentage points. However, this difference had a negligible effect on our simulated policy impacts which were based on comparisons across a much broader range of CD4 counts.

## Discussion

WHO’s September 2015 revised HIV Treatment and Prevention guidelines recommend–for the first time–eliminating CD4 criteria from the decision to start ART, with the goal of expanding the number of patients receiving therapy. South Africa adopted these guidelines September 2016[11]. Based on the experiences of patients in rural South Africa, we estimate that fewer than one in four newly eligible patients will initiate ART within six months, in the absence of additional efforts to encourage uptake. Attrition between HIV diagnosis and ART initiation is high among all patients, but particularly among healthier patients presenting at higher CD4 counts, a phenomenon that has been described elsewhere[10,31]. Generating demand for early treatment and improving testing-to-initiation procedures are critical priorities if South Africa and other nations are to reap the full benefits of test-and-treat.

Our analyses used a regression discontinuity model as a tool for policy simulation. Regression discontinuity designs are traditionally used to identify local causal effects, comparing patients who present just above and below existing treatment thresholds. However, local effects may not be valid further from the threshold. In this paper, we show how a “global” regression discontinuity approach can be used to simulate the impacts of changes in treatment thresholds (including elimination of thresholds) using existing data and additional assumptions. By placing additional structure on the model–namely, the linearity (in logits) of the potential outcome conditional expectation functions–we obtain more plausible projections of the impacts of threshold changes in the context of treatment effect heterogeneity. Our approach provides a framework for thinking about changes to treatment thresholds that may be applicable to other conditions–diabetes, hypertension, etc.–where clinical decision-making is based on threshold rules on continuous biomarkers.

Why do healthy patients not initiate ART? Patients face large financial and time costs to seeking care even where ART is nominally free, with transport and other ancillary costs accounting for about a third of median income in the study area[32]. The clinical benefits of starting ART at high CD4 counts are also relatively modest, compared to the hassle of taking daily medication and potential side effects[3][4]. Patients may perceive that ART is for sick people and that starting ART is an admission of weakness or a sign of being HIV positive[10]. Future studies could examine whether messaging around the prevention benefits of treatment can change this perception and increase uptake of early therapy.

On the supply side, health systems have not always been designed to accommodate patients’ treatment-seeking behaviors, preferences, and psychological barriers such as procrastination. Recent evidence indicates that accelerated initiation procedures (dispensing ARVs on the same day as HIV testing) can increase rates of initiation by up to 36% and 10-month viral suppression by 26%[33]. In prior analysis of our study population, patients who were told they were eligible for treatment and offered ART were far more likely to remain in HIV care than were patients who were told they were not yet eligible for treatment and offered pre-ART care[34]. Modest changes to initiation protocols could substantially improve ART uptake and retention.

Our estimates of ART uptake among eligible patients are low compared to the published literature[35][36]. We emphasize three potential reasons for this difference. First, ART initiation may be underreported, e.g. if persons initiated ART in the private sector or outside of Hlabisa sub-district. We suspect this is a minor explanation. Private sector ART is relatively rare in the study area. Further, by defining our outcome as ART initiation within six months, we limit the influence of outward migration and care-seeking beyond the catchment area.

Second, we assess treatment uptake in a poor, rural area in which the ART program is led by nurses and decentralized to primary care clinics[17]. Outcomes in such a setting–a common model of care in rural southern Africa–may differ from outcomes in well-resourced urban clinics previously described[36–38]. Although levels of ART uptake observed in Hlabisa are lower than in other HIV research cohorts, the proportion initiating ART may be even lower in other non-research settings.

Third, and perhaps most critically, we include all patients from the date blood was taken for their first CD4 count, which is typically the date when the patient tested positive in the clinic. Thus, our denominator includes patients who tested positive but never returned to receive their CD4 count result. Although the inability to disentangle different steps in the pre-ART continuum of care is a limitation, our estimate of aggregate attrition from HIV diagnosis to ART initiation is a highly relevant parameter for assessing the success of the health system in getting patients onto therapy. All patients in our study had contact with the health system, had a CD4 count blood draw, and (for those with CD4<350) were eligible to initiate ART. That many did not go on to initiate ART highlights important gaps and opportunities to intervene.

Our study forecasts the effect of a policy recently implemented, and our estimates necessarily rest on some key assumptions. First, we assume that the distribution of first CD4 counts observed in 2013 will persist into the future. Population increases in HIV testing could shift the distribution of first CD4 counts. As we are unable to anticipate such changes, our results should be interpreted as predictions in the absence of further increases in HIV testing.

Second, we assume that the extrapolated predictions of ART uptake for patients above 350 under revised guidelines represent unbiased estimates of the true (unobserved) counterfactual for these patients. Our preferred specification extrapolates the downward-sloping regression line estimated below 350 cells, and we allow for uncertainty in the slope of the regression line. We also explore the sensitivity of our results to an alternate assumption–that ART uptake in eligible patients was constant above 350 cells–a plausible upper bound. The approach taken here departs from the typical “local” regression discontinuity design, the more common application of regression discontinuity[22,25,39,40]. Whereas continuity in potential outcomes at the threshold is the key identifying assumption in “local” regression discontinuity designs, our “global regression discontinuity” approach assumes that the relationship between first CD4 count and ART uptake is linear (in logits), even in unobserved regions. The reward for this stronger assumption is the ability to estimate treatment effects globally, to describe effect heterogeneity across baseline CD4 counts, and simulate different threshold changes.

Third, we assume that the relationships between first CD4 count and ART uptake (if eligible and if not eligible) are consistent in the future. Though not testable, this assumption is supported by the stability of these functions over time. S2 Fig compares our results for August 2011 –December 2013 with the pattern of ART uptake by CD4 count during January 2007 –August 2011 when the CD4 threshold was 200 cells. The pattern has remained broadly consistent. Nevertheless, there are substantial uncertainties regarding the longer term evolution of the epidemic, care-seeking patterns, and health system response. Our study was designed to capture the short run changes in ART uptake that can be anticipated from eliminating CD4 thresholds. Longer run projections and cost-effectiveness analysis would require feeding our results into dynamic simulation models of the epidemic[12] and the cost of the response[41], an analysis that is beyond the scope of this paper.

Fourth, our projections of national numbers on therapy depend on the assumption that the patterns observed in Hlabisa are similar to patterns that would be observed nationally. This is a strong assumption, but unavoidable given current data limitations. The data required for this analysis–systematic data on pre-ART CD4 counts, linked to ART initiation dates–are not available nationally and are not collected by most other clinical cohorts in South Africa. To the extent that patterns in *f*_*CD4*_, *f*_*ART(1)|CD4*_, and *f*_*ART(0)|CD4*_ deviate in other settings from Hlabisa, these differences easily can be incorporated in our model as new data become available. We note that in newly linked data from South Africa’s National Health Laboratory Services, the distribution of CD4 counts among patients seeking care nationally lags substantially behind the distribution of CD4 counts we observe at presentation in the Hlabisa cohort[42], suggesting that, if anything, our national projections may overestimate the numbers seeking care and starting ART at high CD4 counts.

Finally, our study specifically set out to forecast the impact of eliminating CD4 counts from ART eligibility criteria. It is possible that other changes in initiation guidelines, e.g. same day initiation, could increase uptake of ART. Changes to HIV testing or linkage procedures could both increase the number seeking care and change the characteristics of the patient population. However, as we do not know what future policy will look like, we have specifically assessed ART uptake under the scenario of a limited policy intervention: removal of CD4 criteria, without other changes to treatment protocols.

Our results suggest that as South Africa implements WHO guidance to eliminate CD4 thresholds, the benefits are likely to be modest unless other aspects of the national HIV care and treatment program are also strengthened. Though eliminating thresholds has been proposed as one of the critical pillars to a broader treatment-as-prevention strategy, we estimate that this single step will increase the total number of patients receiving ART in South Africa by 5.2% per year. This pales in comparison to the near doubling of patients on ART required to achieve universal ART coverage in South Africa. While this will benefit many thousands of patients, and is thus of value regardless of other program changes, it is not enough to achieve, on its own, South Africa’s 90-90-90 targets.

## Supporting information

S1 Fig Effect of CD4-count eligibility on ART initiation at the 350-cell threshold: Regression-discontinuity estimate. Dots are proportions estimated within 25-cell bins. Fitted line is a local linear regression with a rectangular kernel and a bandwidth of 97 cells obtained using the Imbens-Kalyaranaman data-driven optimal bandwidth selector. Top-coded data at 999 cells were excluded from the regression fit. Estimates of the predictions at the 350-cell threshold are presented in S1 Table.
(EPS)

S2 Fig Probability of starting ART for patients presenting at different CD4 counts under the 200-cell (pre-Aug 2011) and 350-cell (post-Aug 2011) regimes. Dots are proportions estimated within 25-cell bins. Hollow red dots show the discontinuity in initiation probabilities at 350 cells. Solid blue dots show the discontinuity in initiation probabilities at 200. The initiation probabilities among persons who were always eligible (<200 cells) or never eligible (>350) by CD4 count are similar across the two periods, suggesting that the conditional probability of initiating given CD4 count and eligibility regime may be approximately stable over time, even as the number of patients presenting at different CD4 counts and the eligibility for ART at those CD4 counts changes over time.
(EPS)

S1 Table Regression results: Parameters from logistic and local linear regression models. (DOCX)

S2 Table Sensitivity analysis: Projected impact of guideline changes on numbers of new ART initiators, assuming constant ART uptake above 350 cells under expanded eligibility criteria. (DOCX)
