# Supplementary material for: Effect of eliminating CD4-count thresholds on HIV treatment initiation in South Africa: An empirical modeling study
Source: PLoS One. 2017 Jun 15;12(6):e0178249. doi: 10.1371/journal.pone.0178249 (PMC5472329; doi:10.1371/journal.pone.0178249)
Supplement: S1 Table — (DOCX) [file pone.0178249.s003.docx]

**S1 Table. Regression results: parameters from logistic and local linear regression models.**

| **Parameter estimates** | **Logistic regression**  **(full range of data)** | **Local linear regression**  **(bandwidth = 97 cells)** |
| --- | --- | --- |
| *Regression coefficients* | (in logits) | (in percentage points) |
| $\beta_{0}$*,* intercept | -2.3 (-2.5 to -2.2) | 16.5 (13.5 to 19.5) |
| $\beta_{1}$*,* (CD4-350)*100 | -0.0 (-0.8 to 0.2) | -12.9 (-17.9 to -7.9) |
| $\beta_{2}$*,* 1[CD4<350] | 2.0 (1.8 to 2.1) | 22.9 (18.0 to 27.8) |
| $\beta_{3}$*,* 1[CD4<350]*(CD4-350)*100 | -0.1 (-0.2 to -0.1) | 8.0 (-0.6 to 16.6) |
| *Predicted probabilities at the threshold (*100)* | | |
| Probability of starting ART in 6 months for patients just above the threshold | 8.7 (7.6 to 9.9) | 16.5 (13.5 to 19.5) |
| Probability of starting ART in 6 months for patients just below the threshold | 40.8 (38.8 to 42.9) | 39.4 (35.5 to 43.3) |
| Difference in probability of starting ART just below vs. just above the threshold | 32.1 (29.7 to 34.5) | 22.9 (18.0 to 27.8) |

**Notes:** Table presents regression results from two regression-discontinuity models, each of the form $\Pr\left( ART6mo \right)=\beta_{0}+\beta_{1}\left( CD4-350 \right)+\beta_{2}1\left[ CD4<350 \right]+\beta_{3}1\left[ CD4<350 \right]*\left( CD4-350 \right)$. The local linear regression model was estimated over a window of data +/- 97 cells from the 350-cell threshold, using a data-driven bandwidth obtained using the Imbens-Kalyanaraman optimal bandwidth algorithm. The global logistic regression model was estimated for the full range of the data, 0 to 1000 cells/mm^3^. Predicted probabilities are estimated as $\beta X$ in the linear model and $\exp\left( \beta X \right)/(1+\exp\left( \beta X \right))$ in the logit model.
