# Supplementary material for: Effect of eliminating CD4-count thresholds on HIV treatment initiation in South Africa: An empirical modeling study
Source: PLoS One. 2017 Jun 15;12(6):e0178249. doi: 10.1371/journal.pone.0178249 (PMC5472329; doi:10.1371/journal.pone.0178249)
Supplement: S2 Table — (DOCX) [file pone.0178249.s004.docx]

**Table S2. Sensitivity analysis: projected impact of guideline changes on numbers of new ART initiators, assuming constant ART uptake above 350 cells under expanded eligibility criteria.**

| **Parameter** | **First CD4 count 350 to 500 cells** | **First CD4 count at least 500 cells** |
| --- | --- | --- |
| *Distribution of first CD4 counts in Hlabisa Cohort* |  |  |
| (1) Percent of all patients seeking care in 2013 | 20.0% | 38.9% |
| *Predicted ART uptake in patients presenting with CD4 350-500 and ≥500 cells, Hlabisa Cohort* | | |
| (2) Percent expected to initiate ART even under the old regime, e.g. due to staging | 8.4% | 8.0% |
| (3) Percent expected to initiate ART under expanded CD4 criteria | 40.8% | 40.8% |
| (4) Percent expected to initiate ART due to expanded CD4 criteria, i.e. “new initiators”, (3) – (2) | 32.4% | 32.8% |
| (5) Percent expected not to initiate ART in spite of being eligible under expanded criteria, 100% – (4) | 59.2% | 59.2% |
| (6) Percent increase: ratio of “new initiators” to ART initiators in 2013, (4) * 7973/2233 – 100% | 23.1% | 45.7% |
| *National projections* |  |  |
| (7) Number of additional ART initiators per year nationally expected from expanded CD4 criteria, (6) * 617,000 | 142,000 | 280,000 |
| (8) Percent increase in South Africans receiving ART due to expanded CD4 criteria, (7) / 3 million. | 4.7% | 9.3% |

Table presents sensitivity analyses for our projected impacts of guideline changes extending eligibility for ART to all patients presenting with CD4 counts 350 to 500 cells/mm^3^ and over 500 cells/mm^3^. These calculations assumed a constant proportion of ART uptake (40.8%) above 350 cells under expanded eligibility criteria. *There were 7973 patients who sought care and 2233 ART initiators in 2013 in the Hlabisa cohort. National projections based on 614,000 ART initiators in 2013 and 3 million people currently receiving ART.
